# Diagnosis and treatment of polycystic ovary syndrome (PCOS): An interview with Richard Legro

**DOI:** 10.1186/s12916-015-0299-2

**Published:** 2015-03-27

**Authors:** Richard Legro

**Affiliations:** Penn State University College of Medicine, H103, RM C3604, 500 University Drive, M.S. Hershey Medical Center, Hershey, PA 17033 USA

**Keywords:** Polycystic ovary syndrome, Infertility, Endocrine

## Abstract

In this podcast, we talk to Professor Richard Legro about the recommendations for the diagnosis and treatment of polycystic ovary syndrome (PCOS) based on clinical practice guidelines and discuss the challenges of diagnosis PCOS at specific age groups. The controversies associated with treatment of PCOS, including therapies for infertility as this is a problem commonly observed in PCOS subjects, are highlighted together with future directions on the topic.

The podcast for this interview is available at. http://www.biomedcentral.com/content/supplementary/s12916-015-0299-2-s1.mp3

## Introduction

Richard S. Legro, MD, is Professor in the Department of Obstetrics and Gynecology at Penn State University College of Medicine in Hershey, Pennsylvania, USA. He graduated from Mount Sinai School of Medicine in New York, NY, completed his residency at Magee Womens Hospital at the University of Pittsburgh, and received his fellowship in reproductive endocrinology at the University of Southern California in Los Angeles, CA.

**Figure 1:**
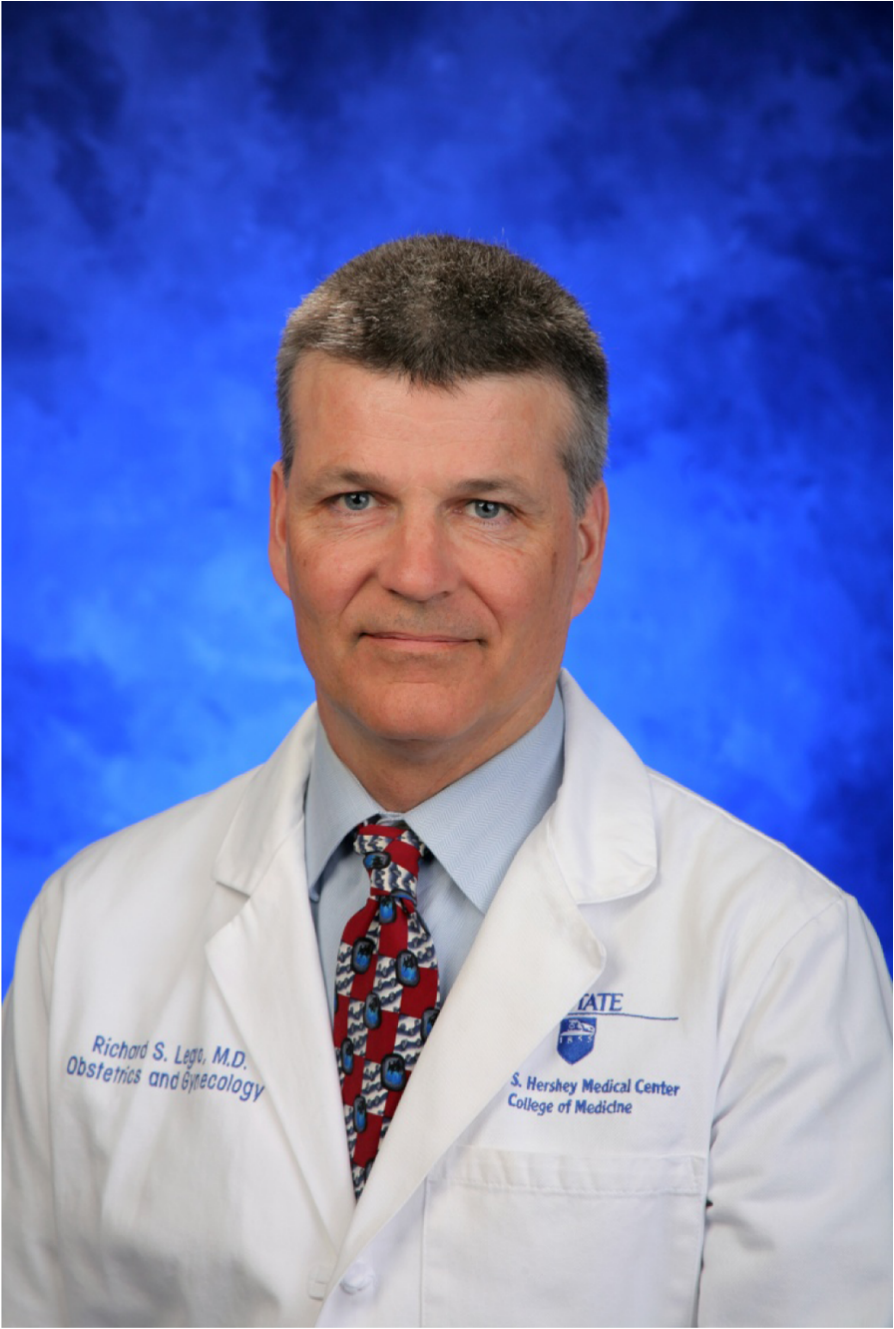
**Richard Legro.**

His research and clinical practice are primarily focused on polycystic ovary syndrome (PCOS) diagnosis, treatment, and genetic/environmental causes. He established one of the first clinics devoted to the treatment of women with PCOS at the M.S. Hershey Medical Center. Dr Legro is currently an investigator in an ongoing Genome Wide Association Study (GWAS) in PCOS and is the lead investigator of the multicenter U.S. National Institutes of Health (NIH)-sponsored trials, Pregnancy in Polycystic Ovary Syndrome I and II. He is the head of the steering committee on several multicenter infertility trials currently underway in China, including PCOSAct, a trial of clomiphene and acupuncture in women with PCOS. He has served as a member of multiple NIH study sections and has published over 200 articles in peer-reviewed journals and books in the field of reproductive endocrinology. He is additionally on the editorial boards for *Endocrine Reviews* and *Endocrinology* and is an Associate Editor for *Seminars in Reproductive Medicine* and *Fertility and Sterility*. He has received several awards including the Presidential Achievement Award from the Society for Gynecologic Investigation (Figure 1).

The edited podcast for this interview is available at: http://www.biomedcentral.com/content/supplementary/s12916-015-0299-2-s1.mp3.

## Edited transcript

### You’re a leading expert in reproductive endocrinology, particularly in PCOS. Can you explain why you decided to specialize in this particular endocrine condition?

This has had a lot to do with my mentors. These included Dr David Guzick where I was a resident in Pittsburgh, and Dr Roger Lobo in Los Angeles with whom I did my fellowship. I subsequently was a junior faculty member here with Dr Andrea Dunaif who is a reproductive and medical endocrinologist who really educated me in all facets of the syndrome. I think what attracted me to it is how little we understand it and how afflicted the patients are. It is a difficult syndrome because we do not have good treatments for a lot of their complaints, but it is rewarding in that every small step we make – either in understanding it or treating it –helps a lot of women, because it certainly is the most common endocrinopathy in the developed world.

### You’ve been a lead author for the Endocrine Society Clinical Practice Guidelines on PCOS that was published in *JCEM* in 2013. As we understand, this provides evidence-based recommendations for the diagnosis and treatment of PCOS. Can you describe the proposals outlined in these guidelines for diagnosing the condition?

We recommended that the so-called Rotterdam criteria should be used to diagnose PCOS. Those criteria are based on finding two of the three cardinal features that characterize PCOS. The first is hyperandrogenism which is androgen excess and diagnosed either on the basis of a clinical sign, such as hirsutism. This includes some midline body hair, primarily on the lip, lower lip, and midline between the breasts and the escutcheon that tracks up to the umbilicus. Hyperandogenism can also be diagnosed on the basis of elevated circulating levels of androgens. The androgen that is most commonly used is testosterone, but androstenedione may also be useful. This is particularly helpful for populations that do not manifest hirsutism. Specifically, this tends to be the Chinese, Japanese and Korean populations that do not have a lot of apparent hirsutism but have evidence of circulating androgen levels comparable to that found in Caucasian cohorts.

In addition to hyperandrogenism, the second is ovulatory dysfunction, and that can be manifested as oligomenorrhea or infrequent menstruation. Sometimes this is observed as secondary amenorrhea or total lack of periods for a while. The third component is a characteristic of the appearance of the ovaries known as the polycystic ovary. It is a misnomer because the ovary really is not polycystic but it contains a lot of very small follicles, that are the size of a quarter inch or less, that develop into ovulatory follicles. This enlarges the ovary with lots of follicles and usually increases the central stoma, a central portion of the ovary that is solid. This appearance of the polycystic ovary is the third sign.

If you have two or three of the conditions indicated above, you qualify for the diagnosis of polycystic ovary syndrome. We relied on a workshop that had been held and sponsored by the National Institutes of Health (NIH), that upheld the expert consensus based Rotterdam criteria. One of the things we wanted to focus more on was treatment rather than diagnosis, so we went with the conventional opinion [for diagnosis]. Everyone should be aware though that there is a lot of controversy on what the proper diagnostic criteria are. Certainly I would say the main criticism of Rotterdam criteria is that it can include subjects who have no hyperandrogenism. Thus, a woman can have irregular periods and polycystic ovaries. It appears that these women have a different prognosis than the women with hyperandrogenism. Specifically, these women tend to have less insulin resistance, less glucose intolerance, less lipid abnormalities, and probably are more likely to be normal weight.

All the criteria agree that you should exclude common conditions that may mimic some of the signs and symptoms of polycystic ovary syndrome. Thus, women should be screened for thyroid dysfunction, prolactin excess, and the onset of non-classical congenital adrenal hyperplasia. In rare cases, where there are signs of virilization or Cushingoid signs, women should be screened for these other causes of severe androgen excess like an androgen-secreting tumor or, in the case of the Cushingoid symptoms, should be screened for Cushing’s Syndrome. Thus, all the guidelines agree that one should exclude conditions that may mimic polycystic ovary syndrome.

### Are there any specific challenges regarding the diagnosis of PCOS associated with specific age groups – so the adolescent or menopausal women?

In regards to the question about the specific challenges associated with adolescent and menopausal women, I think it is difficult or nearly impossible to diagnose it in adolescent and menopausal women. The reason for that is puberty very much mimics the signs and symptoms of polycystic ovary syndrome. Puberty is really characterized by the awakening of the adrenal gland, and that contributes to the formation of body hair in the pelvic region, and also the awakening of the ovary, which results in the completion of puberty, development of secondary sexual characteristics, such as breast development. The initial period is characterized by relative androgen excess, much like polycystic ovary syndrome.

Similarly, it is well known that girls, after they have their first period (termed menarche) can have periods within the first three years of relative oligo-ovulation and infrequent menses. Obviously it varies for different populations; some girls can normalize almost immediately after menarche and other girls have a longer period of ovulatory normalization.

The third characteristic is the polycystic ovary and what really characterizes menarche is the appearance of multiple small antral follicles. It is very easy to confuse a polycystic ovary with what we would call a multi-cystic ovary. In prevalence studies, as many as 50 to 60 percent of normal girls who are teenagers will have what is called a polycystic ovary by current diagnostic criteria. It is very difficult to diagnose this in adolescence because there is a tendency to over diagnose the condition. Additionally, I think part of it is observing and not giving a girl a label that she might not really have. Patience is required, I think, to make this diagnosis. As you know, patience does not characterize 21st century medicine. Thus I think that it is being over diagnosed in adolescents right now.

In terms of menopausal women, I think what is the most difficult thing is recall of menses which is highly inaccurate. A woman who has gone through menopause – for her to recall what her menstrual cycle was like as much as 20 years prior – is difficult. It has been shown that women with PCOS tend to develop more regular menses as they age, and by age I am talking about as they approach their late 30s and 40s, when they appear to have more regular ovulations. Thus, the natural history of polycystic ovary syndrome may be that it resolves in this age range, and certainly during the perimenopause, which is a period of five-to-seven years prior to and surrounding menopause.

The other thing that characterizes menopause is that, much like adolescence, it is a period of relative androgen excess. Thus, with loss of ovarian follicles, there is loss of the production of estrogen by these ovarian follicles, but the ovarian stroma still produces androgen. Thus, menopause *per se* almost qualifies, as every menopausal woman could be diagnosed with PCOS, at least on the basis of biochemical hyperandrogenemia (and cessation of menses). Therefore, it tends to be a diagnosis that I do not think I would make for the first time either in an adolescent or in a menopausal woman.

### We’ve focused on the diagnosis of PCOS. If we move on to the category of treatment of PCOS, can you describe the recommendations made in the Clinical Practice Guidelines that aim to improve treatment?

One of the take-home messages was to focus on the complaint of the patient. The complaints can be variable. If a patient presents with a primary complaint of hirsutism, then one should certainly treat the hirsutism. If the primary complaint is infertility, one should treat the infertility. If the primary complaint is a menstrual disorder, either oligo-ovulation, infrequent bleeding, frequent bleeding or menorrhagia (heavy and prolonged menses), then one should treat that complaint.

Unfortunately, treatments vary according to complaints. To give you an example, for infertility, where we tend to induce ovulation, there is really no treatment that induces ovulation that also adequately treats hirsutism. Thus, we often have to put hirsutism on the back burner while we treat infertility. Similarly, most treatments for hirsutism involve suppression of the ovaries and suppression of any possibility for ovulation. It is difficult to primarily treat hirsutism and secondarily hope for resumption of ovulation fertility.

Thus, several of the clinical practice guidelines focus on what many of us feel is the *sine qua non* of the syndrome, which is hyperandrogenism. The best treatment for female hyperandrogenism is some form of hormonal suppression. This would include hormonal contraception – obviously birth control pills are used the most commonly – but there is also a contraceptive patch and a vaginal ring. There are also contraceptive formulations that are primarily a progestin and injections and implants of progestin can be used to suppress ovarian hyperandrogenism.

### That’s really interesting in terms of the variability in treatment. Are there any controversies associated with the treatment of PCOS?

I would say that the primary controversy is one which goes back to my training with a medical endocrinologist and reproductive endocrinologist and one that still has not been sorted out. This controversy is on what extent polycystic ovary syndrome is a metabolic disorder. If the women have been shown to be insensitive to the effects of insulin, they often compensate with over-secretion of insulin. Clearly in the periphery, the skeletal muscle is very resistant to the action of insulin. This certainly predisposes them to Type 2 diabetes and metabolic syndrome and, questionably, to cardiovascular diseases. Certainly, they have increased cardiovascular disease risk.

The other school of thought says that polycystic ovary syndrome is primarily a reproductive disorder with inappropriate communication of the brain and pituitary with the ovary. If one can get these systems in sync you can have a normal ovulatory woman.

Thus, depending on which school of thought you approach this with – either the metabolic or reproductive schools – then the appropriate therapies should be chosen. The reproductive school certainly recommends the use of hormonal contraceptives to suppress hyperandrogenism, or the use of oral agents that affect estrogen action – so primarily clomiphene, which is an estrogen-receptor inhibitor – to treat infertility. The metabolic school focuses more on trying to improve insulin action. Metformin tends to be the first choice by that school for many complaints, both from a metabolic aspect as well as treating hyperandrogenism. Metformin does lower circulation androgens and improves ovulation rate. Many use it also as a fertility drug. However, it does not appear to be as good as clomiphene or letrozole (an aromatase inhibitor).

Another approach is lifestyle modification, with an emphasis on weight loss as a means of improving the metabolic picture. I think one of the things we outlined in the guideline, and also in two meta-analyses that were published concurrently in the same year in the *Journal of Clinical Endocrinology and Metabolism*, was that we found that, while certainly lifestyle therapy was associated with improvements in glycemic parameters like weight, there was not much that we could say about hyperandrogenism. While certainly, we did not want to go against any recommendations that an obese woman should undergo lifestyle modifications to improve her diabetes and cardiovascular risk, it is difficult to say that that is going to improve their PCOS symptoms. Here, PCOS is primarily defined on the basis of reproductive parameters.

Similarly with metformin, much like lifestyle improves glycemic parameters, it lowers testosterone, but is probably weight neutral in most studies. It is hard to advocate this as an effective first-line treatment for the primary complaints that these women present with: hirsutism, infertility and menstrual disorder. Metformin just does not work as well as, for instance, the oral contraceptive pill or oral ovulation induction agents like clomiphene or letrozole. Thus, metformin probably should take a back burner compared to the other treatments in the guideline. This has created a lot of controversy and we did our best to defend our recommendations and I think we did.

### Clearly, these are comprehensive guidelines for the treatment and diagnosis of PCOS and you have touched upon this earlier – how it compares with the Rotterdam criteria and the NIH recommendations – but how also does it compare with previous Endocrine Society ones?

When the Endocrine Society does develop guidelines, we carefully review other guidelines. There were many other guidelines that were relevant to this, including the treatment of hirsutism, the amelioration of cardiovascular risk factors and the diagnosis of Cushing’s disease. Thus, we referenced these other guidelines in ours and we do not really tread on their territory; we defer to them and echo any of their diagnostic recommendations. This, however, was the first Endocrine Society guideline for polycystic ovary syndrome. We were inventing the wheel, at least the polycystic ovary syndrome wheel.

### You also mentioned earlier about fertility as a problem in women with PCOS, so let us discuss the recent trial that you published in *NEJM*. This trial compares the use of letrozole and clomiphene for infertility in PCOS. Can you briefly describe the findings of that trial and how you think this might inform future therapies for infertility in women with PCOS?

We performed a double-blind multi-center controlled trial, testing letrozole versus clomiphene for infertility in women with polycystic ovary syndrome. The hypothesis was that letrozole (an aromatase inhibitor) might be superior for the outcome of live birth. Again, that is what patients who are infertile want: a live birth. We thought an aromatase inhibitor might recruit fewer follicles than clomiphene. Clomiphene is an infertility drug associated with an eight-to-ten-fold increased risk of multiple pregnancies. Certainly from a public health perspective, given the morbidity – especially from pre-term delivery of multiple pregnancies – this was definitely an outcome we wanted to avoid. There also were data showing that an aromatase inhibitor might have more favorable effects on the endometrium and aid implantation after ovulation.

In our trial, we showed that women who used letrozole had a 44 percent greater chance of a live birth compared to clomiphene. This is a fairly significant difference. It is actually about a 10 percent absolute increase in the pregnancy rate in women who had undergone our treatment, which was five cycles of treatment with either clomiphene citrate or letrozole. Indeed, while not significant, we found that the multiple pregnancy rate was 7.4 percent with clomiphene and 3.9 percent with letrozole. This was a secondary outcome and, while it was not significant, one needs a trial that was probably about five times as big to detect that difference in rate. However, we are optimistic that lowering the multiple pregnancy rate might be a beneficial side-effect of letrozole.

However, one of the concerns about letrozole was that there might be an increased risk of birth defects, compared to clomiphene. One of the things that has been well documented in sub-fertile women is that they do have a slightly higher rate of birth defects than women who conceive spontaneously. We found that the rates of birth defects were comparable between letrozole and clomiphene and, actually, both were below what we would expect in an infertile population. Thus, we were reassured about that. However, like any trial like this which we think will change our practice, replication of these results is needed. Clomiphene has been really the first line treatment since the 1960s, so this was, I think, rather a remarkable finding.

### As we now draw to the close of this interview, can you just tell us what you think are the most promising future directions for diagnosing and treating PCOS?

Certainly in diagnosis, there has been a lot of research trying to look at an endocrine serum marker that might identify women with a tendency towards polycystic ovary syndrome. Probably the most promising serum marker is a hormone called anti-Müllerian hormone (AMH), which roughly correlates with a number of small antral follicles. Thus, this may be a useful blood test especially in younger women where an ultrasound may be unwanted and invasive and one that all practitioners could use, not just gynecologists who have easy access to trans-vaginal ultrasound. I think the jury is still out on that and we have to see more. Another promising marker is our genetic markers that are being identified through genetic studies and genome-wide association studies. I think we have to validate these markers prospectively, and that work is being done.

In terms of the treatment of PCOS, I think there is a lot of interest now in developing oral drugs that might perturb the way the brain and the pituitary communicate with the ovary. I think a lot of these drugs may be very useful for the treatment of polycystic ovary syndrome.

Another big question and one that I am writing up the results right now – is what to do with obese women with PCOS who want to get pregnant? How much should we focus on lifestyle modification and weight loss prior to treatment and how much we should just proceed directly to treatment. We really need prospective trials that test a lifestyle modification versus a controlled alternate treatment to see what the benefits might be in terms of improving pregnancy outcomes in both mother and infant.

### Where can I find out more?

See references [1-10].
